# Development of a novel photocatalyst for the advanced antibiotic oxidation of wastewater

**DOI:** 10.1186/2193-1801-4-S2-O7

**Published:** 2015-11-27

**Authors:** Dickson Yuk-shing Yan, Frank Leung-yuk Lam

**Affiliations:** Faculty of Science and Technology, Technological and Higher Education Institute of Hong Kong, Hong Kong; Department of Chemical and Biomolecular Engineering, The Hong Kong University of Science and Technology, Hong Kong

## Background

Between 2000 and 2010, the consumption of antibiotics in 71 countries increased by 36% [1]. The rise of antibiotic consumption is expected to continue, considering the potential genetic mutation of super bacteria. Most antibiotics cannot be fully metabolised by the human body [2] and are excreted into the environment via wastewater effluents. Typical biological treatment processes, which have been used for several decades in wastewater treatment, normally require a long processing time for the development of new cells in biomass. More importantly, antibiotics are not easily degraded by bacteria, so the removal of antibiotics from wastewater is of only low efficiency. Researchers have therefore recently attempted an advanced oxidation processes (AOP) for the treatment of antibiotics in wastewater. In addition to the widely used photocatalyst TiO_2_, bismuth oxybromide, BiOBr, has received much attention in recent years for its generation of hydroxyl radicals, due to its high stability and low band gaps. This study aims to modify BiOBr for enhancing the oxidation of antibiotics. Success in this study will bring a new insight to the understanding of catalysis.

## Methods

BiOBr nanoparticles were synthesised using a hydrothermal method. Chelant-modified solutions of citric acid (CA) and ethylenediaminetetraacetic acid (EDTA), were added separately in the course of BiOBr preparation. In addition, an Au/Ag-deposited BiOBr composite was prepared using photodeposition with different volumes of HAuCl_4_ and AgNO_3_ solutions. Kinetic batch experiments of norfloxacin photodegradation were carried out under UV irradiation. The concentration of norfloxacin was measured by high-performance liquid chromatography and its organic content was measured by total organic carbon analysis.

## Results

The chelating-agent modified BiOBr displayed superior photodegradation of norfloxacin under UV irradiation (about 70% removal) compared to BiOBr without modification. A molar ratio of CA/Bi = 0.5 was found to be more effective on norfloxacin degradation than on EDTA. Significant degradation of norfloxacin (about 70% removal) was achieved with the bismuth-based catalyst with 1% Au or Ag by weight. An increase of metal deposition posed a negative effect on norfloxacin degradation.

## Conclusions

The effectiveness of norfloxacin removal can be enhanced by BiOBr modification with CA, EDTA and nano Au/Ag. To improve the photoactivity of BiOBr for sustainable removal of antibiotics, BiOBr should be further modified for effective oxidation of various types of antibiotic under visible light spectrum. From an engineering perspective, the ultimate goal is to develop a pilot-scale efficient photocatalytic system for antibiotic oxidation. With the highly competitive manufacturing base in South China, Hong Kong could take a leading position in the environmental control devices market without enormous capital investment. A new photocatalytic system will significantly improve outdoor water quality, which is a critical issue for urbanised countries, and draw attention to the long-term sustainability of environmental research Hong Kong.
